# Supplementary material for: WordCommentsAnalyzer: A windows software tool for qualitative research
Source: F1000Res. 2018 Sep 4;7:536. Originally published 2018 May 3. [Version 2] doi: 10.12688/f1000research.14819.2 (PMC6137411; doi:10.12688/f1000research.14819.2)
Supplement: Supplementary file 3 [file f1000research-7-17614-s0002.tgz › b2a79496-4ada-47c4-9682-a99ce867836d.zip!/Eight Iranian Health Journals/Salmand_Iranian Journal of Ageing _47.docx]

© Salmand_Iranian Journal of Ageing . This work is licensed under CC BY-NC 4.0 (https://creativecommons.org/licenses/by-nc/4.0/).

Volume & Issue: دوره 1 - شماره 1

Title: بررسی وضعیت سلامت سالمندان در شهرستان کاشان

http://salmandj.uswr.ac.ir/article-1-26-fa.html

اهداف:‌ جهت دستیابی به سلامت سالمندان و پیشگیری از بروز و ابتلای آنان به بیماریهای مزمن و کاهش شیوع بیماریهای ویژه در آنان اولین گام شناسایی وضعیت سلامت آنان است. هدف از این مطالعه تعیین وضعیت سلامت سالمندان شهرستان کاشان مشتمل بر وضعیت سلامت روانی، وضعیت سلامت شناختی، وضعیت تعادل و راه رفتن، وضعیت تغذیه و وضعیت خطر ابتلا به زخم بستر به منظور هدف کاربردی پیشگیری در جامعه و تعیین نیازهای بهداشتی و درمانی سالمندان گروه بوده است. مواد و روش‌ها: پژوهش حاضر یک تحقیق توصیفی مقطعی می‌باشد. جامعه پژوهش مشتمل بر کلیه افراد 55 سال و بالاتر موجود در خانواده هایی که در سال 1383 در شهرستان کاشان زندگی می‌کنند، می‌باشد. روش نمونه‌گیری تصادفی ساده بود. برای جمع‌آوری اطلاعات از پرسش‌نامه استفاده شد که مستقیما با مراجعه پرسش‌گر به سالمند نسبت به تکمیل آن اقدام گردید. یافته‌ها: یافته ها نشان داد که (41.7درصد) بیماری های عضلانی اسکلتی و قلبی عروقی شایع ترین مشکل سالمندان می باشد. (41.7درصد). بر اساس یافته ها، 68.3درصد از سالمندان شهرستان کاشان در معرض خطر ابتلا به سوء تغذیه، 30درصد تغذیه طبیعی و 5.8درصد مبتلا به سوء تغذیه می‌باشند. همچنین 34.2درصد جمعیت مورد بررسی، افسردگی شدید و 44.1درصد افسردگی متوسط داشته و 21.7درصد افسردگی نداشتند. بر اساس یافته ها، 5درصد از سالمندان مورد بررسی، اختلال شناختی شدید، 47.5درصد اختلال شناختی متوسط، 30درصد اختلال شناختی خفیف داشته و 17.5درصد اختلال شناختی نداشتند. انجام فعالیت های روزانه در 81.7درصد از سالمندان خوب (کاملا مستقل)، 13.3درصد متوسط (نیازمند به کمک) و 5درصد کاملا غیرمستقل و برای انجام کارهای روزانه به دیگران وابسته هستند و نهایتا بر اساس نتایج 3.3درصد از افراد در معرض خطر ابتلا به زخم بستر بودند. نتیجه‌گیری: از آنجایی که بسیاری از بیماری های دوران سالمندی به راحتی قابل پیشگیری و کنترل است، لذا توصیه می‌گردد جمعیت شهرستان در مورد پیشگیری، آزموش های لازمه را دریافت دارند. موضوعات و اولویت های پیشنهادی آموزشی با توجه به نتایج تحقیق عبارت‌اند از: نحوه پیشگیری از ابتلا به بیماری های عضلانی استخوانی، قلبی عروقی، زخم بستر و آموزش های لازمه در مورد تغذیه و سبک صحیح زندگی می‌باشد که می‌تواند به صورت کارگاه‌های آموزشی، بولتن و پمفلت آموزشی جامعه هدف را مورد خطاب قرار دهد.

Title: بررسی اعتبار، روایی، تحلیل عامل مقیاس سلامت عمومی (GHQ) فرم 28 سؤالی در سالمندان ایرانی

http://salmandj.uswr.ac.ir/article-1-27-fa.html

اهداف: به دلیل روند روبه رشد جمعیت سالمند حتی در کشورهای در حال رشد، وجود اختلالات روانی در آنان از مشکلت بهداشتی‌درمانی این کشورها محسوب می‌گردد. بدین منظور وجود ابزارهای غربالگری هنجارشده برای انجام مطالعات همه‌گیرشناسی و بالینی ضروری است. هدف اصلی این تحقیق ساخت نسخه ایرانی فرم 28 سوالی مقیاس سلامت عمومی (GHQ) در سالمندان بود. مواد و روش‌ها: نسخه فارسی پرسشنامه با مطابقت ترجمه و ترجمه معکوس آن تهیه شد. 204 سالمند ساکن شهرک اکباتان تهران به‌صورت تصادفی انتخاب شده و این مقیاس تکمیل شد. مصاحبه ساختار یافته CIDI به عنوان ملاک استاندارد برای تعیین تشخیص آزمودنی‌ها طبق ملاک های DSM-IV انجام گرفت. یافته‌ها: در فرم ایرانی تغییراتی در طبقه‌بندی سوالات ایجاد شد و ضرایب پایانی مطلوبی اعم از آلفای کرونباخ (0.94)، تنصیف (0.86)، آزمون-بازآزمون (0.6) به‌دست آمد. تحلیل عوامل چهار عامل «افسردگی»، «عملکرد اجتماعی»، «اضطراب» و «علایم جسمانی» را مشخص نمود. با انجام آزمون ROC، با حساسیت 0.83 و ویژگی 0.76 بهترین نقطه برش 19.20 به‌دست آمد. فرم کوتاه 14 سوالی از فرم اصلی استخراج گردید. نتیجه‌گیری: فرم‌های 28 و 14 سوالی مقیاس سلامت عمومی از پایانی و اعتبار مطلوبی برای استفاده در مطالعات همه‌گیرشناسی و بالینی در جمعیت سالمند به‌ویژه در مناطق شهری برخوردارند. لازم است اعتباریابی فرم 14 سوالی در مطالعات مستقل مورد ارزیابی قرار گیرد.

Title: وضعیت آگاهی رابطان بهداشت شهرستان شیراز در خصوص شیوه زندگی سالم در دوره سالمندی

http://salmandj.uswr.ac.ir/article-1-28-fa.html

اهداف: با توجه به روند روبه افزایش جمعیت سالمندان، ترویج بهداشت سالمندی و ارتقا سطح سلامت سالمندان به عنوان یک اولویت بهداشتی مطرح است که باید برای آن برنامه‌ریزی مناسب انجام شود. بسیاری از مشکلات دوران سالمندی ناشی از شیوه زندگی ناسالم در این دوران است. لذا باید آموزش شیوه زندگی سالم و مراقبت‌های بهداشتی این دوران به جامعه به‌ویژه سالمندان مورد توجه خاص قرار گیرد. یکی از راهکارهای مناسب در زمینه ترویج آموزش، استفاده از رابطان بهداشتی است که با همکاری آنان می توان به آسانی آموزش لازم را به خانوارها خصوصا سالمندان انتقال داد. با توجه به اهمیت آموزش و نقش رابطان بهداشتی، این مطالعه به منظور تعیین میزان آگاهی رابطان بهداشت شهرستان شیراز انجام گردید. مواد و روش‌ها: این مطالعه به صورت مقطعی به منظور تعیین سطح آگاهی رابطان بهداشت در زمینه شیوه زندگی سالم در دوره سالمندی در سال 84 انجام گردید. در این مطالعه 320 نفر از رابطان بهداشتی شهرستان شیراز به طور تصادفی انتخاب شدند. حجم نمونه با استفاده از نتایج پایلوت بر روی 100 نفر از رابطان در این مورد محاسبه گردید. اطلاعات لازم با استفاده از یک پرسش‌نامه تهیه شده مربوط به خصوصیات دموگرافیک افراد و سوالات در زمینه شیوه زندگی سالم و مراقبت‌های لازم در دوران سالمندی با روش مصاحبه حضوری جمع‌آوری گردید. نتایج حاصله با استفاده از نرم‌افزار EPI2002 کدگذاری و با تست‌های آماری توصیف شد. یافته‌ها: طبق نتایج به‌دست آمده میانگین سطح آگاهی رابطان بهداشت در زمینه مراقبت‌های بهداشتی دوران سالمندی 66.9 درصد و میانگین آگاهی آن‌ها در رابطه با شناخت و پیشگیری از بیماری‌های شایع سالمندی 66.3 درصد بود. نتیجه‌گیری: نتایج مطالعه حاضر نشانگر ضرورت آموزش شیوه زندگی سالم به جامعه و نقش مشارکت مردمی به منظور گسترش سطح آموزشی و حرکت به سوی سالمندی سالم و فعال می‌باشد.

Title: خودکارآمدی و فشار مراقبتی مراقبان عضو خانواده سالمندان مبتلا به آلزایمر در تهران

http://salmandj.uswr.ac.ir/article-1-29-fa.html

اهداف: پژوهش حاضر یک مطالعه توصیفی‌تحلیلی از نوع همبستگی است که با هدف کلی تعیین خودکار آمدی و فشار مراقبتی و بررسی ارتباط آن‌ها در مراقبین عضو خانواده سالمندان مبتلا به آلزایمر مقیم در منزل شهر تهران، در سال 1384 انجام پذیرفته است. مواد و روش‌ها: در این مطالعه 81 نفر از مراقبین عضو انجمن آلزایمر ایران بودند. ابزار جمع‌آوری داده‌ها پرسش‌نامه مشتمل بر اطلاعات فردی؛ سنجش خود کار آمدی و فشار مراقبتی مراقبین عضو خانواده بود. یافته‌ها: یافته‌ها نشان داد اکثریت واحدهای مورد مطالعه را زنان 60 درصد، با نسبت همسری 56 درصد، متاهل 64 درصد، در سطح تحصیلات سیکل 39 درصد، خانه‌دار 55 درصد که با سالمند تحت مراقبت در یک خانه به سر می‌برند (52درصد) تشکیل می‌داد. 45 درصد این واحدها برای ارائه مراقبت آموزش رسمی نداشتند و 53 درصد نیز از ارایه مراقبت به سالمند خود راضی بودند این در حالی بود که 36 درصد آنان سلامت خود را بد ارزیابی نموده و 35درصد نیز مدعی ابتلا به اختلالات روان بودند. مهم‌ترین نیاز مراقبتی مراقبین را نیاز به آموزش تشکیل دادته و از نظر آنان اولین اولویت مراقبت‌دهی به سالمندانشان حمایت و مراقبت روحی و روانی، و به ترتیب در درجات بعدی حمام دادن و رعایت نظافت بیمار قرار داشت. میانگین خودکارآمدی 66.96 (106-29) و فشار مراقبتی 39.43 (65-17) محاسبه شد. این دو متغیر با هیچ‌یک از متغیرهای منظور در مشخصات فردی مرتبط نبود و بین دو متغیر همبستگی معنی‌دار 0.539 با P=0.01 برقرار بود. نتیجه‌گیری:‌ یافته‌ها نشان داد که دو متغیر خودکارآمدی و فشار مراقبتی مفاهیمی کاملا فردی بوده و به برآورد و ادراک کلی فرد از خود بستگی دارد. از سوی دیگر اگر چه مراقبت از سالمند مبتلا به آلزایمر امری استرس‌آور محسوب می‌شود. لیکن ارزیابی مراقبین از این استرس به گونه‌ای نیست که مراقبین، آن را غیر قابل رویارویی تعبیر کنند. به‌طوری‌که میانگین در حد متوسط فشار مراقبتی و ارتباط مثبت آن با احساس خودکارآمدی نشان‌دهنده تعبیر بالنسبه مثبت مراقبین از استرس مراقبت می‌باشد. یافته‌های این مطالعه با یافته سایر محققین در حوزه مراقبت همخوان بود.

Title: بررسی تأثیر برنامه آموزش بهداشت تغذیه بر کارکنان آسایشگاه کهریزک شهر تهران در ارتقای سلامت تغذیه‌ای سالمندان مقیم آن آسایشگاه

http://salmandj.uswr.ac.ir/article-1-30-fa.html

اهداف: سالمندی به عنوان دوره‌ای از زندگی تلقی می‌گردد که با تغییراتی در متابولیسم و سوخت‌و‌ساز بدن همراه است. این تغییرات سالمندان را مستعد بروز سوء تغذیه می نماید، چنانچه طی بررسیها 13-3درصد از سالمندان جامعه مبتلا به سوء تغذیه هستند و این میزان در سالمندان ساکن آسایشگاه بین 60-30درصد است. در این مطالعه تاثیر آموزش به کارکنان پزشکی‌بهداشتی و مراقبان سالمندان بر وضعیت تغذیه‌ای سالمندان مقیم آسایشگاه کهریزک شهر تهران در مدت چهار مورد ارزیابی قرار گرفت. مواد و روش‌ها: 192 نفر سالمند از سالمندان علاقمند به ورود به مطالعه (50 مرد و 142 زن) ساکن در آسایشگاه خیریه کهریزک که 65 سال و بیشتر سن داشتند و به بیماری‌های کبدی و کلیوی پیشرفته مبتلا نبودند و سابقه عمل جراحی در یک ماه قبل از شروع مطالعه را نداشتند، به طور تصادفی انتخاب و مورد بررسی قرار گرفتند. در این افراد آزمایشات هموگلوبین، هماتوکریت، لیپوپروتیین کم تراکم سرم، لیپوپروتیین پرتراکم سرم، قند خون ناشتا و C.R.P قبل و پس از مداخله اندازه‌گیری گردید. همچنین ابزارهای بررسی وضعیت تغذیه MNA، بررسی وضعیت مختصر شناختی MMSE، بررسی وضعیت ADL و بررسی خطر زخم بستر Norton توسط کارشناسان تغذیه ارزیابی گردید. سپس تمام کارکنان و مراقبان پزشکی‌بهداشتی آسایشگاه کهریزک به مدت سه روز در یک کارگاه آموزشی تحت آموزش قرار گرفتند و پس از گذشت چهار ماه مجددا تمام آزمایشات پاراکلینیکی و تست‌های بالینی فوق انجام گردید. بعد از ورود اطلاعات، تجزیه‌وتحلیل آماری با نرم‌افزار آماری SPSS انجام گرفت. یافته‌ها: به‌طور کلی بر اساس یافته‌های حاصل از این تحقیق و بر اساس ابزار بررسی MNA، 35/9درصد از سالمندان قبل از آموزش دچار سوء‌تغذیه بسیار شدیده بوده اند که این میزان پس از آموزش به 18/3درصد کاهش یافته است. پس از آموزش میانگین آلبومین از 3/98 به 4/11، هموگلوبین از 12/7 به 13/03درصد، هماتوکریت از 37/86 به 38/8 و LDL از 135/13 به 146/25 و F.B.S از 81/92 به 95/8 افزایش یافت و اختلاف معنی‌دار بود. میانگین HDL از 43/84 به 42/85 رسید و اختلاف معنی‌دار نبود. وضعیت تغذیه با سن، اختلال در دهان، اختلال در بلع، مصرف داروهای مورد نظر، وضعیت شناختی افراد، وضعیت امتیاز ADL افراد در غذاخوردن و امتیاز نورتون، رابطه معنی‌دار داشت. نتیجه‌گیری: براساس یافته‌های حاصل از پژوهش می‌توان نتیجه گرفت که آزمایش در بهبود شاخص‌های تغذیه‌ای ازجمله آلبومین، هموگلوبین و هماتوکریت موثر بوده و در کاهش تعداد افراد با سوء‌تغذیه شدید و متوسط نیز موثر بوده است. پیشنهاد می‌گردد، برنامه‌های آموزشی تغذیه به‌صورت مستمر و همراه با مداخله در رژیم غذایی افراد، ارائه گردد.

Title: اختلالات گفتار و زبان در سالمندان

http://salmandj.uswr.ac.ir/article-1-31-fa.html

اهداف: از آنجایی که پدیده سالمندی با توجه به افزونی جمعیت آن در ملل گوناگون روبه افزایش است، لذا در حال حاضر اغلب کشورهای توسعه‌یافته و یا در‌حال‌توسعه جهان توجه به اهمیت برنامه‌های مختلف اجتماعی و اقتصادی، سالمندی را در برنامه‌های توسعه خود جای داده‌اند. مواد و روش‌ها:‌ با توجه به افزایش شمار سالمندان در کشورمان، توجه خاص به نیازهای مختلف آن‌ها مستلزم برنامه‌های تدوین‌شده‌ای است که با تغییرات آناتومیکی، نوروآناتومیکی و فیزیکی این گروه ارزشمند جامعه مطابقت داشته باشد. یافته‌ها: حفظ توانایی‌های کیفی و کمّی گفتار و زبان در دوره سالمندی می‌تواند نقش ارزشمندی در ارتباط آن‌ها با جامعه داشته باشد. هر چقدر توانایی برقراری ارتباط در آن‌ها بیشتر باشد سایر معضلات حاشیه ای که ممکن است به دنبال این عدم ارتباط حاصل گردد نیز کم می‌گردد. نتیجه‌گیری: آگاهی و شناخت پدیده سالمندی و تاثیر این پدیده در کلیه اجزا مولد «گفتار» و «زبان» برای عموم درمانگران، خصوصا آسیب‌شناسان گفتار و زبان حایز اهمیت است. با بررسی اولیه مناسب و تشخیص نوع اختلال گفتار و زبانی سالمند، می‌توان برنامه درمانی مناسبی را آغاز نمود که این درمان و مداخله زود هنگام می‌تواند موجب ارتقای همه‌جانبه ارتباط سالمند با دنیای خارج شود.

Title: سوء‌ مصرف مواد در سالمندان

http://salmandj.uswr.ac.ir/article-1-32-fa.html

سوءمصرف مواد به‌خصوص مواد افیونی و داروهای تجویزشده توسط پزشکان، به سرعت در حال گسترش میان سالمندان است. بسیاری از اوقات مصرف مواد به منظور رفع تنهایی، تسکین دردهای مزمن و ضعف در سلامت جسمانی آغاز می‌شود. در موارد دیگر، سوءمصرف مواد افیونی از دوران جوانی به سالمندی کشیده می‌شود. به‌هرحال، متاسفانه این مشکل کمتر مورد توجه  متخصصان و دست‌اندرکاران امور اعتیاد و سالمندی قرار گرفته است. به‌طوری که هیچ اطلاعی از شیوع این اختلال، مشکلات همراه با آن و مداخلات پیشگیرانه و درمانی موثر در دوران سالمندی در دست نیست. این مقاله ضمن تاکید بر لزوم برنامه‌ریزی برای شناسایی و درمان سوءمصرف مواد در سالمندان، تعدادی از روش‌های فوق درمانی در کشورهای دیگر را معرفی و مرور می‌نماید.

Title: مروری بر مکانیسم‌ ها و تئوری‌ های پیری

http://salmandj.uswr.ac.ir/article-1-33-fa.html

فاکتورهای متعددی مشوق مطالعات مربوط به طول عمر و افزایش حداکثر طول عمر و افزایش درصد سالمندان جوامع و افزایش درصد بودجه جوامع که صرف سالمندان می‌شود از آن جمله‌اند. دیدگاه‌های اخیر نسبت به مکانیسم‌های پیری حاکی از فرایندهای بسیار پیچیده و چندعاملی می‌باشد که برخلاف دیدگاه‌های اولیه است که بر علت مشخص مانند اثر یک ژن یا یا از بین رفتن یک عامل کلیدی تاکید می‌کردند. این مرور مختصر سعی دارد مکانیسم‌های پیری را در سطوح ملکولی سلولی و سیستمیک بررسی نماید و همچنین جنبه‌ای مختلف تاثیر متقابل ژنتیک و محیط را مدنظر داشته باشد. تئوری‌های تکاملی پیری را نتیجه کاهش نیروی انتخاب طبیعی می‌دانند و در مقابل تئوری‌های مولکولی بر تنظیم ژنتیکی پیری تاکید دارند و سالمندی را نتیجه تغییرات در ژن می‌دانند. تئوری‌های سلولی نیز وجود دارند که مشهورترین آن‌ها تئوری تلومر است و پیری حاصله از استرس نیز در این گروه قرار دارد. تئوری‌های سیستمیک قرار دارند که شامل دور گروه اصلی نورواندوکرین و ایمونولوژیک است.
